# The Yin and Yang of protein folding

**DOI:** 10.1111/j.1742-4658.2005.05021.x

**Published:** 2005-12-01

**Authors:** Thomas R. Jahn, Sheena E. Radford

**Affiliations:** Astbury Centre for Structural Molecular Biology and Institute of Molecular and Cellular Biology, Gerstang Building, University of Leeds, UK

**Keywords:** amyloid fibril formation, energy landscapes, intermediates, misfolding, protein folding

## Abstract

The study of protein aggregation saw a renaissance in the last decade, when it was discovered that aggregation is the cause of several human diseases, making this field of research one of the most exciting frontiers in science today. Building on knowledge about protein folding energy landscapes, determined using an array of biophysical methods, theory and simulation, new light is now being shed on some of the key questions in protein-misfolding diseases. This review will focus on the mechanisms of protein folding and amyloid fibril formation, concentrating on the role of partially folded states in these processes, the complexity of the free energy landscape, and the potentials for the development of future therapeutic strategies based on a full biophysical description of the combined folding and aggregation free-energy surface.

## Introduction

**Fig. 1:**
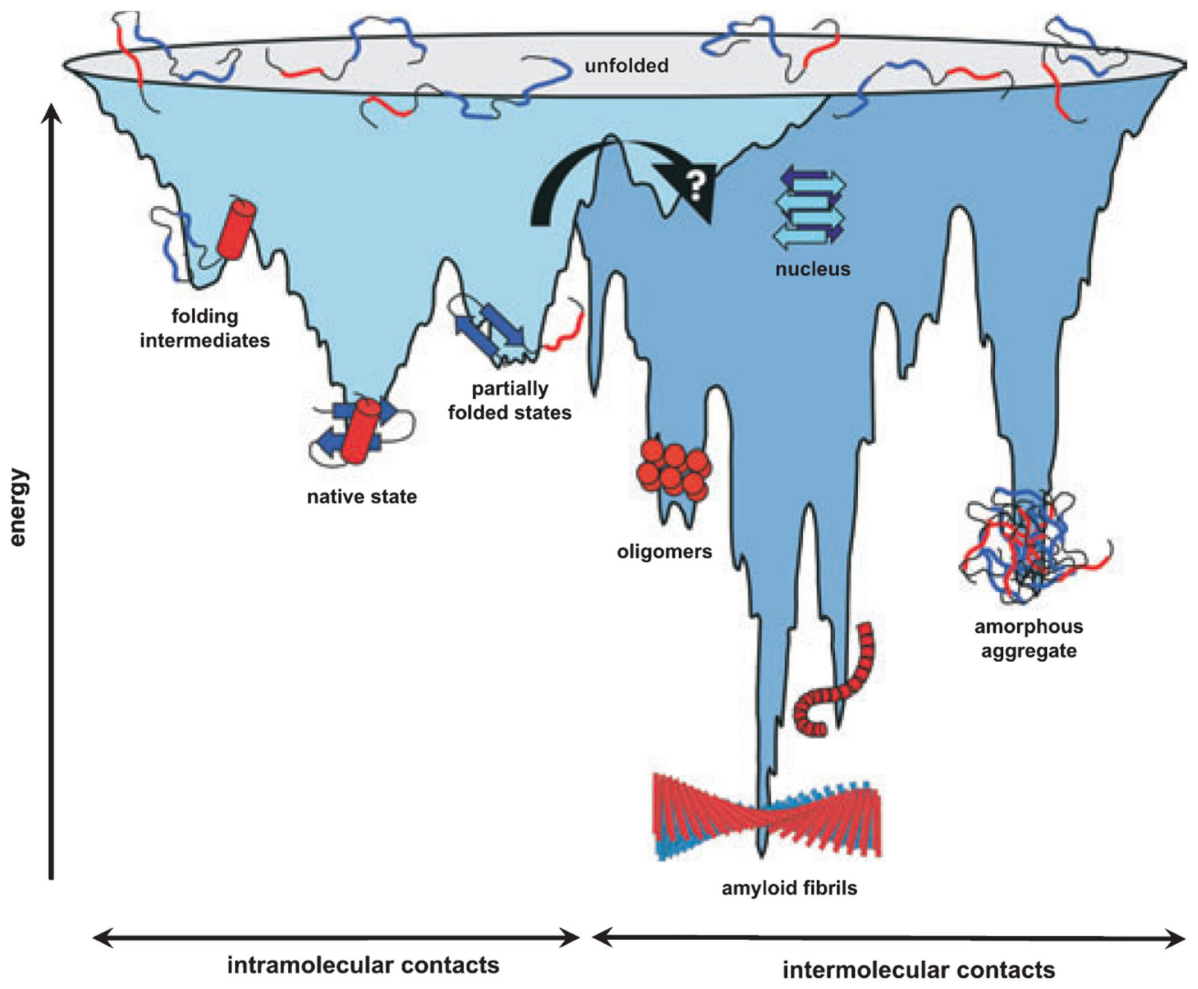
A schematic energy landscape for protein folding and aggregation. The surface shows the multitude of conformations ‘funneling’ towards the native state via intramolecular contact formation, or towards the formation of amyloid fibrils via intermolecular contacts. Recent experiments have allowed the placement of different ‘intermediate’ structures on both pathways [2,50], although detailed structural models for many of these species are not yet available. Furthermore, the species involved in converting kinetically stabilized globular structures into the thermodynamic global free energy minimum in the form of amyloid fibrils for different proteins is currently not defined.

**Fig. 2:**
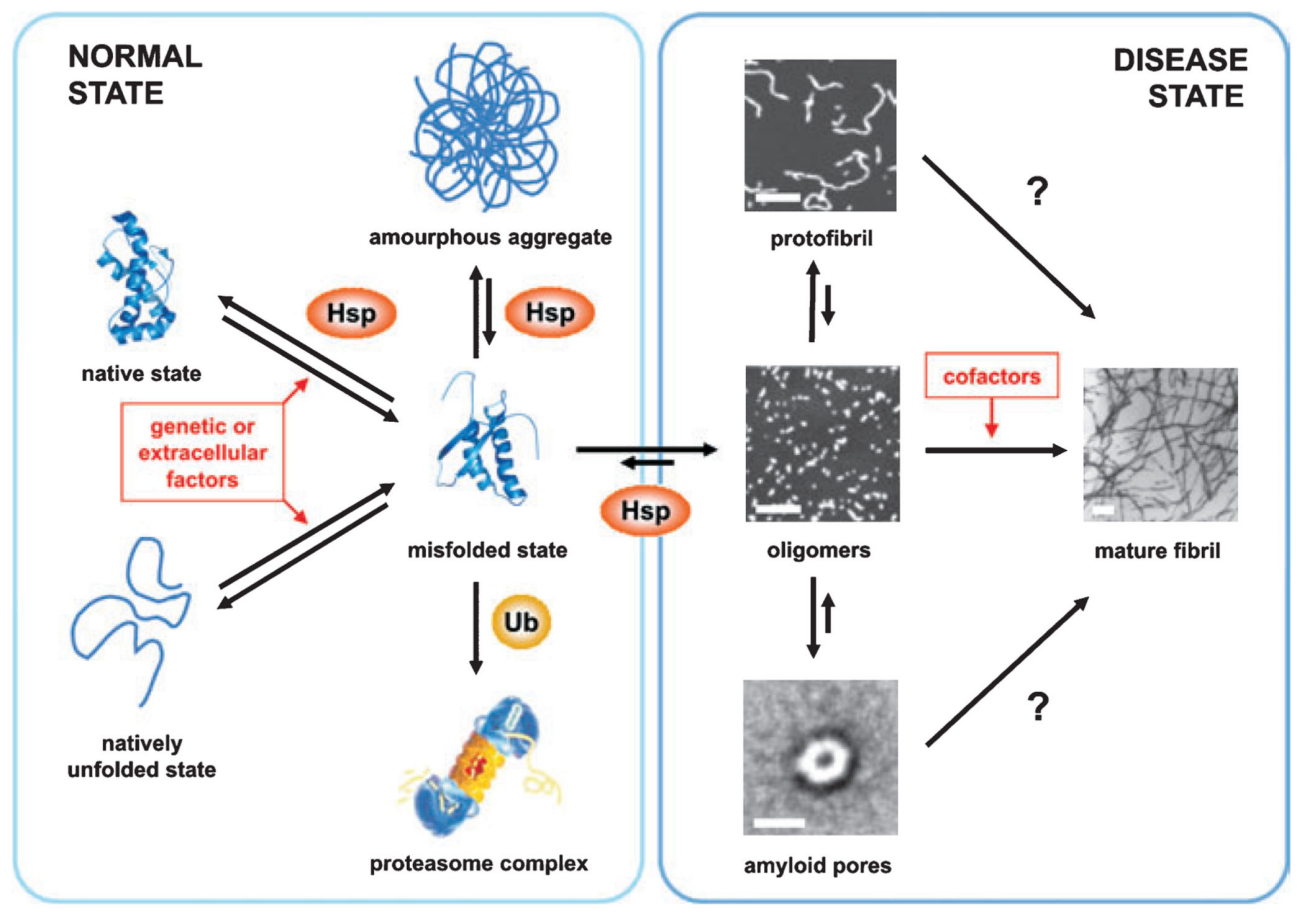
A schematic representation of the factors influencing protein folding and aggregation events *in vivo*. Molecular chaperones (Hsp) as well as the ubiquitin-proteasome pathway (Ub) prevent protein unfolding and aggregation by facilitating refolding or degradation, respectively. An increased population of misfolded proteins as a result of genetic or extracellular factors may lead to a saturation of these defense mechanisms and subsequently to an increase in protein aggregation. Partially folded proteins associate with each other to form small, soluble oligomers that may undergo further assembly into protofibrils, oligomeric pores or mature fibril deposits (scale bars represent 100 nm or 10 nm for the amyloid pore) [37,38]. Whether these species can interconvert, or whether the indicated structures represent assembly end products, is dependent on the assembly conditions and the identity of the polypeptide sequence [38,50]. The toxicity of different species and their role in the development of disease is currently being explored for different protein systems [39].

The ability of proteins to fold *de novo* to their functional states is one of the most fundamental phenomena in nature. Since the pioneering work of Anfinsen and co-workers [1], numerous studies of protein folding have been carried out, and major insights into the nature of protein-folding mechanisms, including structural, kinetic and thermodynamic analyses of intermediates and transition states, from experiment, theory and simulation, are now emerging [2]. Currently, energy landscapes are used to describe the search of the unfolded polypeptide down a funnel-like energy profile towards the native structure (Fig. 1). The surface of this folding funnel is unique for a specific polypeptide sequence under a particular set of conditions and is determined by both thermodynamic and kinetic properties of the folding polypeptide chain. Partially folded states on this landscape may be intrinsically prone to aggregation, and favorable intermolecular contacts may lead to their association and ultimately to protein-misfolding diseases (Figs. 1 and 2). The mechanisms underlying these specific aggregation events has drawn intense interest in the protein-folding community in recent years, as this has expanded the impact of studies of protein folding from a key fundamental question to a central issue in the understanding of several human diseases. One of the most commonly studied classes of protein aggregation disorders is amyloid disease. In these disorders, amyloid fibrils are found as deposits of insoluble aggregates, accumulating in patients with a range of maladies including Alzheimer’s and Parkinson’s diseases, type II diabetes and Creutzfeldt–Jacob disease [3]. In this review we describe current knowledge about the energy landscapes of protein folding and protein aggregation, and highlight the need to study both mechanisms in detail to understand how they are connected. We then discuss recent insights into the structural properties of folded and partially folded species and describe the role of these states in the folding energy landscape in the context of amyloid fibril formation. Finally, we describe current concepts of how non-native states can assemble in such a specific manner into the ordered cross-β structure of amyloid and discuss how cellular rescue mechanisms may help to shape the folding and aggregation energy landscapes *in vivo* to facilitate folding to a functional form, whilst preventing aggregation.

## Protein folding energy landscapes

Historically, protein folding was considered as a series of sequential steps between increasingly native-like species, until the final native structure is formed. Based on the realization that the unfolded and partially folded states are conformationally heterogeneous, and that there may not be a single route to the native state, models of folding have now evolved into the landscape view of protein folding [4], in which the unfolded polypeptide chain searches for the native conformation on a usually rugged energy surface or ‘landscape’, until the unique native structure is formed (Fig. 1). Random fluctuations in the unfolded or partially folded states drive this reaction, as different native as well as non-native contacts are sampled. In general, native interactions between residues are assumed to be more stable than non-native contacts, and as such contacts form, the number of available conformations is reduced, driving the polypeptide chain towards the native structure.

Small single domain proteins (e.g. < 100 amino acids in length), in general, fold to the native state on a sub-second timescale and have been the focus of many experimental and theoretical studies of folding [5]. The folding landscape of these proteins is usually relatively smooth, resulting in only two species being stably populated during the folding reaction – the ensemble of unfolded structures and the native state – separated by a single transition state barrier (i.e. these proteins fold with a two-state mechanism) [6]. The very rapid and efficient search is encoded by a network of interactions between ‘key residues’ in the structure, forming a folding nucleus that establishes the native topology in the transition state ensemble (the folding transition bottleneck) [7]. In the case of the 98-residue protein, acylphosphatase, Vendruscolo and co-workers determined that as few as three residues are sufficient to determine the topology of this α/β protein [8]. Delineating the mechanism of folding has resulted in the development of a plethora of exciting experimental approaches (Table 1), from measurements of folding on nano- to microsecond timescales [9] to single molecule experiments [10]. In addition, protein engineering methods (monitoring the effect of amino acid substitutions on the kinetics of folding and unfolding) have been shown to be unique in their ability to probe the role of individual residues in stabilizing the structure of partially folded intermediates, as well as high-energy transition states [11]. Theoretical studies, particularly involving simulation techniques, have been used to complement experimental data, and vice versa, allowing a complete view of folding from the earliest steps to conformational transitions as the native structure ultimately forms [12,13].

Proteins larger than ≈ 100 residues in length fold on a much rougher energy surface in which folding intermediates are commonly populated en route to the native state. The reason for this seems to be that larger chains have a higher tendency to collapse in aqueous solvent, resulting in the formation of compact states that may contain substantial elements of native-like structure. Reorganization of interresidue contacts (including both native and non-native interactions) in these compact states may involve a high free-energy barrier, leading to the transient population of partially folded or ‘intermediate’ states (Fig. 1). Such species can be productive for folding (on-pathway), or trapped such that the native structure cannot be reached without substantial reorganizational events (the intermediate is off-pathway). There is ongoing discussion about whether intermediates assist folding by limiting the search process, or whether they are traps that inhibit rapid folding [14], and evidence for both abounds [15,16]. In large multidomain proteins, parallel folding of different regions allows their independent topological search, while a final folding step establishes all native intra- and interdomain contacts that define the final functional form [17], possibly picturing the sequential folding events on the ribosome *in vivo* [18].

Since the advent of modern multidimensional NMR methods and X-ray crystallography, we have learned much about the structure and dynamics of proteins in their native conformations. On the other hand, the conformational properties of unfolded proteins and intermediate states are more difficult to define, as their heterogeneity, complexity and rapid interconversion rules out detailed structural analysis at high resolution by these methods. However, recent NMR approaches, involving relaxation measurements, residual dipolar couplings and hydrogen exchange, combined with molecular dynamics simulations using these, and other, parameters as constraints, are beginning to cast light on the structural properties of different ensembles on the folding energy landscape [19,20].

## Mechanisms of protein misfolding and aggregation

A large number of protein-misfolding diseases belong to a class of grave human disorders known as ‘amyloidosis’ [3], because the aggregated protein forms so-called ‘amyloid fibrils’ that can be stained with the dye Congo red in a manner similar to starch (amylose) [21]. One of the striking characteristics of this class of diseases is that the associated proteinaceous fibrils are very similar in their overall properties and appearance, forming a cross-β structure in which continuous β-sheets are formed with β-strands running perpendicular to the long axis of the fibril [22,23]. This structure is remarkable, not just in its commonality, stability and insolubility, but also because the precursor proteins that comprise the fibrils have no sequence similarity and are structurally very diverse, ranging from small peptides [amyloid β-peptide (Aβ), amylin, insulin], through natively unfolded proteins (α-synuclein), to natively folded monomeric proteins [lysozyme, β_2_-microglobulin (β_2_m)] or even protein assemblies [transthyretin (TTR)]. Most intriguingly, these amyloidogenic proteins have native structures that are virtually indistinguishable from their nonamyloidogenic native counterparts [3], which, together with the observation that many proteins not known to be involved in amyloid disease can aggregate *in vitro* into amyloid-like structures, strongly suggests that the formation of the cross-β fold is an inherent property of the polypeptide chain [24]. Therefore, understanding the mechanism of fibril formation for one protein may also cast important insights on how all proteins can assemble into the beautiful, yet deadly, structure of amyloid [25].

Studies of the structural transition between soluble precursors and insoluble amyloid fibrils have recently become possible, as amyloid formation can be induced *in vitro*, opening the door to detailed mechanistic analysis using the techniques developed to monitor protein folding (Table 1). In the case of globular proteins, fibrils typically form under conditions in which the native state is destabilized (i.e. by the addition of denaturant, low pH, high temperature or amino acid substitutions), with the result that the population of the partially folded conformations is increased [26]. Partial unfolding is essential, as the native states of these proteins are not amyloidogenic (Fig. 2). Which factors cause destabilization of the native structure and the increase in the steady-state concentration of partially folded conformers *in vivo* is now becoming clear for some proteins involved in amyloid disorders [27]. In the case of the enzyme lysozyme, the aggregation of which is involved in hereditary systemic amyloidosis, single point mutations in the lysozyme gene are associated with fibril deposition in several tissues. Two amyloidogenic variants have been studied in detail and were shown to be significantly less stable than the wild-type protein and, importantly, also lack the cooperativity of the native structure, leading to an increased concentration of partially folded states at equilibrium [28]. The same principle applies for TTR variants involved in familial amyloidotic neuropathy. Thus, amyloidogenic TTR variants have been shown to have a decreased tetramer stability and an increase in the tetramer dissociation rate constant that, together, lead to an increase in amyloidogenesis [29]. Therefore, for these proteins, alterations in the amino acid sequence increase their amyloid propensity. For other proteins, changes in the local environment or the concentration of wild-type protein can result in the onset of amyloid disease. For example, β_2_m forms amyloid deposits in the disorder dialysis-related amyloidosis [30]. For this protein, the full-length wild-type protein is the aggregating sequence. Two factors are known to be important in the development of amyloid for β_2_m, (a) an increased serum concentration (up to 60-fold) owing to renal impairment (the normal site of β_2_m catabolism), and (b) a decreased stability of the monomeric protein compared with its major histocompatibility complex (MHC) class-I bound counterpart. Finally, for unfolded proteins such as α-synuclein, partial folding has been shown to be an essential first step in self-assembly [31], underlining the importance of partially folded species as amyloid precursors. However, the identity of the specific amyloid precursor structure has not yet been determined for any protein, resulting in a currently missing link between the folding and aggregation funnels (Fig. 1).

## Sculpting the energy surface *in vivo*

In the living cell, a large machinery of proteins forms the quality-control system, ensuring the correct folding of proteins on one hand, and the rapid degradation of mutated or misfolded polypeptides on the other [32,33] (Fig. 2). The folding of newly synthesized proteins to their native conformations involves the sequential action of multiple molecular chaperones [33,34]. Two major chaperone classes, Hsp70 and Hsp60, act in a tightly controlled ATP-dependent manner to bind and release unfolded or misfolded substrates, thereby enhancing substrate refolding and preventing aggregation [33]. Furthermore, recognition of abnormal proteins by the cellular machinery leads to their ubiquitinylation and subsequent degradation by the 26S proteasome [35] (Fig. 2). However, even for proteins that fold successfully to their native state and hence escape the cellular quality control machinery, random conformational fluctuations can lead to the transient formation of aggregation-prone intermediate states (Fig. 1). In the crowded environment of the cell, and also influenced by environmental factors, such species may then start to aggregate, forming small oligomers or larger particles that initiate the amyloid cascade. Especially in age-related amyloidosis, this may lead to the accumulation of large quantities of partially folded proteins and the saturation of the capacity of the quality control machinery, exacerbating the formation of intracellular aggregates before refolding or degradation is possible [36] (Fig. 2). Recent *in vitro* studies, using electron mucroscopy and atomic force microscopy, have identified and characterized several intermediate structures populated during fibril formation, including small oligomers, membrane embedded pores and protofibrils, the latter having a characteristic ‘beaded’ appearance (Figs. 1 and 2). Whether these structures form on-pathway or are an off-pathway product of fibril formation, and which of these structures are actually the toxic ones, are probably the most debated questions today [37–39]. An exciting study by Stefani and co-workers showed the ‘inherent toxicity’ of these early aggregates, whilst later fibrillar species appear to lack toxicity, suggesting that the fibrillar inclusions may serve a protective role [40]. Most importantly, the proteins used in this study were not naturally amyloidogenic, highlighting that toxicity may be a generic feature of these prefibrillar states. In a recent study, Muchowski and co-workers have shown that the cellular chaperones Hsp70 and Hsp40 attenuate the formation of spherical and annular oligomers, whilst favoring the formation of fibrillar species [41], rationalizing the finding that these chaperones also suppress neurodegeneration in animal models for Huntington’s and Parkinson’s diseases [42]. Even through chaperones like Hsp104 can resolubilize microaggregates, mechanisms for the solubilization and degradation of large proteinaceous deposits are currently poorly understood [43].

As the identity and structural characterization of the toxic species for many amyloid diseases remain unknown, generic approaches for the prevention of toxicity in amyloidosis are still in their infancy [44]. However, attractive therapeutic approaches are based around the idea of smoothing the protein landscape, to prevent the accumulation of aggregation-prone or toxic species. *In vitro* studies of TTR, for example, have shown that small molecules, mimicking the binding of natural ligands, stabilize the native tetrameric structure by binding at the interface between subunits, thereby preventing their dissociation that is known to be a critical first step in the onset of aggregation [45]. Dobson and co-workers used a single-domain fragment of a camelid antibody to rescue the amyloidogenic lysozyme variant, D67H, from amyloid fibril formation [46]. Interestingly, this was achieved by increasing protein stability and restoring the cooperativity between the two structural domains in the native protein, reducing the number of global unfolding events and decreasing the probability of subglobal unfolding and the consequent formation of partially unfolded states. While the properties of the native proteins are encoded by the amino acid sequence, amyloid deposition depends strongly on a number of cofactors, including serum amyloid P, apolipoprotein E and glucosaminoglycans, which bind and stabilize the fibrillar state [47]. In the absence of these factors, fibrils can be rapidly depolymerized, offering another route for therapeutic intervention [48,49]. A clear understanding of the mechanism of the association of these cofactors with amyloid fibrils may expose further possibilities of targeting amyloid deposition, presuming that this does not result in an increase in the production of toxic species.

## Folding vs. aggregation: kinetic partitioning

Amyloid fibrils are formed in a nucleation-dependent manner, in which the protein monomer form is converted into a fibrillar structure via a transient aggregation nucleus [50]. Whilst the structural mechanisms of nucleation and elongation are currently unknown, the residues key to the aggregation process are thought to be different from those important in driving correct folding of the polypeptide chain [51], although the major driving forces (the formation of hydrogen bonds and the burial of hydrophobic surface area) are the same for both processes. Although a large part of the polypeptide chain may be involved in the fibril structure, it is clear that some amino acid sequences are more prone to aggregation than others, as shown by a variety of studies of peptide assembly into amyloid-like fibrils *in vitro* [52]. Thus, akin to a protein-folding reaction, where only a few residues define the folding nucleus, but many, if not all, residues are required to support the structure of the folding transition state [5], key residues may also be important in driving the assembly of the entire polypeptide chain into amyloid fibrils. From a systematic analysis of more than 50 protein variants, Chiti *et al*. rationalized the propensities of some sequences to aggregate more rapidly than others, based on the physicochemical characteristics of the polypeptide chain, namely hydrophobicity, secondary structure propensity and charge [53]. Furthermore, based on similar principles, Serrano and co-workers have developed a generic algorithm, TANGO, that predicts which particular polypeptide sequences will aggregate, rationalizing specific point mutations found in amyloid diseases [54]. Proteins may also have evolved features to prevent aggregation while folding, by introducing ‘negative-folding determinants’. For example, proline residues frequently found in membrane α-helices are thought to maximize correct folding by preventing misfolded (β-sheet) conformations [55]. In addition, the edge strands of native β-sheets are protected from forming intermolecular hydrogen bonds by a number of ‘positive design’ features that protect exposed edge strands from improper intermolecular interactions [56].

The ability of proteins to fold rapidly to their globular ‘native’ structure allows them to escape aberrant side-reactions that would give access to the aggregation funnel and lead to the thermodynamic ground state of intermolecular assembly, the amyloid fibril. Evolution therefore must have shaped the folding and aggregation funnels to allow kinetic trapping of the native functional state, which is thermodynamically a ‘metastable’ structure in the context of the entire protein landscape *in vivo* [57]. Chaperones play an active role in accelerating protein folding by decreasing the roughness of the energy landscape, such that aggregation-prone intermediates are effectively funneled towards the native state. Such a role for the molecular chaper-one, GroEL, has been observed experimentally [58,59] and recently mimicked through molecular dynamics simulations [60]. However, proteins do not exist to fold rapidly into a solid structure, but must fulfill a functional role, leaving the need for dynamical events, of which transient partial unfolding is a natural part. Native proteins thus are only marginally stable relative to the denatured state, and partially folded states can be formed from the folded structure by local or subglobal unfolding events. For most proteins, however, the cooperativity of the protein folding process, and the assistance of the cellular rescue machinery, help to avoid population of partially folded forms (Fig. 2). Changes in the amino acid sequence, alterations in the folding conditions, or breakdown of the cellular control system allows the shift towards the aggregation funnel, whereupon the polypeptide chain folds and assembles into the thermodynamically stable fibril conformation. In a recent study, Kelly and co-workers showed that even small differences in the endoplasmic reticulum machinery can shape folding and assembly, with the result that tissue specificity, severity and the age of onset of extracellular amyloid diseases can be altered significantly [61].

One of the key questions currently unanswered is at which point the folding and aggregation landscapes meet (i.e. whether the separation between the different fates occurs at the unfolded state or whether partially folded forms are also a common entity). Of course, a common mechanism is not required for all polypeptide sequences, and for some sequences the identity of the amyloid precursor may differ under different conditions. To address these questions, the development of techniques used to unravel the characteristics of the folding funnel (Table 1) will be of direct benefit in exploring the conversion of transiently populated states into aggregated structures, although unraveling the heterogeneity of the system will be a significant challenge. As with kinetic studies of folding, molecular dynamics simulations will undoubtedly play an important role, as such techniques are now beginning to be used to probe the conformational conversion of amyloid peptides [62], as well as the docking of precursor units into a final fibril structure [63]. The most fundamental questions about the nature and frequency of different unfolding events, the structural properties of different ensembles, the barrier heights between them and the shape of the multidimensional landscape, are still to be defined.

## Conclusions

In this review we have highlighted the relevance of protein (un)folding in amyloid fibrillogenesis, as the increased population of partially folded states formed by conformational fluctuations from the native state leads to amyloid fibril formation. Although evolution has shaped the protein folding funnel (via changes in the amino acid sequence and the introduction of chaperones, for example) such that partially folded states which are prone to aggregation are only transiently formed, alterations to the protein sequence or a decrease in the effectiveness of the cellular protective mechanisms can dramatically affect the energy landscape, switching from a kinetically favored native, functional state towards the globally most stable structure, the amyloid fibril. The intellectual input from over half a century of experiments on protein folding, structure and dynamics provides a strong platform from which to unravel the structural molecular mechanism of amyloid formation, simultaneously unraveling the cause of debilitating human disease. An advanced knowledge about toxic states populated on the aggregation pathway may subsequently lead to new possibilities of treatment and/or prevention of amyloid disease. The general concept of the multiplicity of protein folding and assembly landscapes discussed in this review may stimulate the development of new ideas and experiments to understand the fundamental driving forces behind these structural transitions, leading to a deeper understanding, not only of polypeptide structure and dynamics, but also of the mechanism of human disease.

## Figures and Tables

**Table 1:** Experimental approaches to characterize protein folding and protein aggregation free energy landscapes^a^. A, amyloid fibril; N, native state; O, small oligomer; U, unfolded or partially folded states.

Experiment	Technique	Species
Kinetic^b^		
Folding/Assembly	Spectroscopy^c^ (absorption, fluorescence, CD, etc.)	U, N, O, A
	NMR (realtime, relaxation and line-shape analysis, etc.)	U, N
	Mass spectrometry	U, N, O, A
	Single molecule experiments (FRET, optical tweezers, etc.)	U, N
	Protein engineering (phi-value analysis, etc.)	U, N
	Specific dye binding (ANS, Thioflavin T, ligands, etc.)	U, N, O, A
	Hydrogen-deuterium exchange	U, N, O, A
	Turbidity and light-scattering	N, O
	Chemical cross-linking	O, A
Equilibrium		
Structure	X-ray crystallography	N
	Fibre diffraction	A
	Solution NMR	U, N
	Solid state NMR	O, A
	Cryo-electron microscopy	A
Conformation	Spectroscopy (see above)	U, N, O, A
	Electron and atomic force microscopy	O, A
	Analytical ultracentrifugation	U, N, O
	Gel permeation chromatography	U, N, O
	Calorimetry	U, N
Dynamics	NMR (relaxation measurements, dipolar couplings, etc.)	U, N
	Hydrogen-deuterium exchange	U, N, O, A
	Denaturant and proteolysis stability	U, N, O, A

a A more detailed description of specific methods can be found (e.g. [64]).

b The most suited species currently analysed using a specific technique are shown.

c Dependent on the time range, methods include manual mixing, stopped flow, continuous flow and relaxation techniques (temperature jump, flash photolysis, etc.).

## Abbreviations

β_2_m: β_2_-microglobulin
TTR: transthyretin
